# Worse recovery from acute attacks and faster disability accumulation highlights the unmet need for improved treatment in patients with late-onset neuromyelitis optica spectrum disorders (COPTER-LO study)

**DOI:** 10.3389/fimmu.2025.1575613

**Published:** 2025-04-11

**Authors:** Julian Reza Kretschmer, Daria Tkachenko, Tania Kümpfel, Joachim Havla, Daniel Engels, Friedemann Paul, Patrick Schindler, Judith Bellmann-Strobl, Achim Berthele, Katrin Giglhuber, Clarissa Zappe, Luisa Klotz, Lisa Revie, Eva Dawin, Makbule Senel, Hayrettin Tumani, Florian Then Bergh, Clemens Warnke, Markus Kraemer, Annette Walter, Antonios Bayas, Uwe K. Zettl, Ann-Sophie Lauenstein, Yavor Yalachkov, Thorleif Etgen, Matthias Kaste, Felix Luessi, Stefan Gingele, Sarah Passoke, Martin S. Weber, Jörn Peter Sieb, Axel Haarmann, Patrick Oschmann, Veit Rothhammer, Christian Geis, Markus C. Kowarik, Peter Kern, Matthias Grothe, Heike Stephanik, Klemens Angstwurm, Frank Hoffmann, Ulrike Wallwitz, Brigitte Wildemann, Sven Jarius, Jan-Patrick Stellmann, Thivya Pakeerathan, Carolin Schwake, Ilya Ayzenberg, Ingo Kleiter, Katinka Fischer, Orhan Aktas, Marius Ringelstein, Vivien Häußler, Corinna Trebst, Martin W. Hümmert

**Affiliations:** ^1^ Department of Neurology, Hannover Medical School, Hannover, Germany; ^2^ Institute of Clinical Neuroimmunology, LMU University Hospital, Ludwig-Maximilians-University Munich, Munich, Germany; ^3^ Department of Neurology, Charite Universitätsmedizin Berlin, Berlin, Germany; ^4^ Charité Universitätsmedizin Berlin, Experimental and Clinical Research Center and Max Delbrueck Center for Molecular Medicine, Berlin, Germany; ^5^ Department of Neurology, Technical University Munich, Munich, Germany; ^6^ Department of Neurology, University of Münster, Münster, Germany; ^7^ Department of Neurology, University of Ulm, Ulm, Germany; ^8^ Department of Neurology, University of Leipzig, Leipzig, Germany; ^9^ Department of Neurology, Faculty of Medicine and University Hospital Cologne, University of Cologne, Cologne, Germany; ^10^ Department of Neurology, Alfried Krupp Hospital Essen, Essen, Germany; ^11^ Department of Neurology, Herford Hospital, Herford, Germany; ^12^ Department of Neurology, Medical Faculty, University of Augsburg, Augsburg, Germany; ^13^ Department of Neurology, University of Rostock, Rostock, Germany; ^14^ Department of Neurology, DKD Helios Hospital Wiesbaden, Wiesbaden, Germany; ^15^ Department of Neurology, Goethe-University Frankfurt, University Medicine, Frankfurt am Main, Germany; ^16^ Department of Neurology, Klinikum Traunstein, Traunstein, Germany; ^17^ Department of Neurology, Nordwest-Krankenhaus Sanderbusch, Sanderbusch, Germany; ^18^ Department of Neurology, University of Mainz, Mainz, Germany; ^19^ Department of Neurology, University of Göttingen, Göttingen, Germany; ^20^ Department of Neurology, Helios Hanseklinikum Stralsund, Stralsund, Germany; ^21^ Department of Neurology, University of Würzburg, Würzburg, Germany; ^22^ Department of Neurology, Klinikum Bayreuth, Bayreuth, Germany; ^23^ Department of Neurology, FAU Erlangen-Nuremberg, Erlangen, Germany; ^24^ Department of Neurology, University of Jena, Jena, Germany; ^25^ Department of Neurology & Stroke and Hertie-Institute for Clinical Brain Research, Eberhard-Karls University of Tübingen, Tübingen, Germany; ^26^ Department of Neurology, Asklepios Expert Clinic Teupitz, Teupitz, Germany; ^27^ Department of Neurology, University of Greifswald, Greifswald, Germany; ^28^ Department of Neurology, University of Magdeburg, Magdeburg, Germany; ^29^ Department of Neurology, University of Regensburg, Regensburg, Germany; ^30^ Department of Neurology, Hospital Martha-Maria, Halle, Germany; ^31^ Molecular Neuroimmunology Group, Department of Neurology, University of Heidelberg, Heidelberg, Germany; ^32^ Institut für Neuroimmunologie und Multiple Sklerose (INIMS), Universitätsklinikum Hamburg-Eppendorf (UKE), Hamburg, Germany; ^33^ CEMEREM, APHM, Hôspital de la Timone, Marseille, France; ^34^ CRMBM, Aix Marseille Univ, CNRS, Marseille, France; ^35^ Department of Neurology, St.Josef Hospital, Ruhr University Bochum, Bochum, Germany; ^36^ Marianne-Strauss-Klinik, Behandlungszentrum für Multiple Sklerose Kranke, Berg, Germany; ^37^ Department of Neurology, Medical Faculty, Heinrich-Heine-University Düsseldorf, Düsseldorf, Germany; ^38^ Department of Neurology, Center for Neurology and Neuropsychiatry, LVR-Klinikum, Heinrich-Heine-University Düsseldorf, Düsseldorf, Germany

**Keywords:** NMOSD = neuromyelitis optica spectrum disorder, aging, late onset, immunoscenecence, myelitis

## Abstract

**Objective:**

This study analyzed clinical characteristics, attack recovery and long-term disability accumulation in late-onset (LO ≥ 50 years at onset) versus early-onset (EO < 50 years) NMOSD.

**Methods:**

This multicenter cohort study included demographic and clinical data from 446 NMOSD patients collected from 35 German Neuromyelitis Optica Study Group (NEMOS) centers. Time to disability milestones was estimated through Kaplan-Meier analysis and Cox proportional hazard regression models adjusted for sex, year of onset, immunotherapy exposure and antibody status. Generalized estimating equations (GEE) were used to compare attack outcomes.

**Results:**

Of the 446 NMOSD patients analyzed (83.4% female, 85.4% AQP4-IgG-positive, median age at disease onset = 43 years), 153 had a late-onset (34.3%). AQP4-IgG+ prevalence was higher in LO- than in EO-NMOSD (94.1% vs. 80.9%, *p*<0.001). Optic neuritis at onset was more frequent in EO-NMOSD (27.4% vs. 42.6%, *p*<0.002), whereas myelitis was more common in LO-NMOSD (58.4% vs. 37.9%, *p*<0.001). Both groups had similar annualized attack rates (AAR, 0.51 vs. 0.54, *p*=0.352), but attack recovery was poorer (complete remission in 15.6% vs. 27.4%, *p*<0.001) and relapse-associated worsening (RAW) was higher in LO-NMOSD (RAW: 3 vs. 0.5, *p*<0.001). Long-term immunotherapy use was comparable. LO-NMOSD exhibited faster progression to disability endpoints (EDSS 4: HR = 2.64, 95% CI=1.81–3.84).

**Interpretation:**

LO-NMOSD patients presented more often with myelitis, experienced worse attack outcomes and faster disability accumulation, despite comparable AAR, acute attack treatment and long-term treatment regimens. Accordingly, therapeutic strategies for attack and prophylactic treatment in LO-NMOSD have to be improved.

## Introduction

Neuromyelitis optica spectrum disorders (NMOSD) are neuroinflammatory diseases commonly characterized by optic neuritis, transverse myelitis, or area postrema syndrome (1). The median age of onset of the disease is approximately 40 years (2). However, NMOSD can also occur at or after the age of 50 and is then classified as late-onset (LO-NMOSD) (3). It may even manifest at advanced age beyond 75 years which is associated with less favorable outcomes (4). Several smaller studies have already investigated age-related discrepancies in NMOSD (**Supplementary Table 1**). These studies have shown that patients with an early onset (EO-NMOSD) of the disease are prone to present with optic neuritis at initial manifestation, whereas transverse myelitis occurs more often in patients with LO-NMOSD (5–11). Furthermore, patients with a late onset of the disease tend to have a higher risk of experiencing severe motor disability, worse visual outcomes and disease-related death (12–14). Accordingly, the time to reach Expanded Disability Status Scale (EDSS) scores greater than 4 was higher in LO-NMOSD (7, 15). Nevertheless, due to small sample sizes and the predominance of Asian study populations, some of which also included patients with myelin oligodendrocyte glycoprotein antibody-associated disease (MOGAD), there are still inconsistencies across the studies, prompting the need for confirmation in larger cohort studies with a focus on older patients. Particularly, attack outcomes, attack treatments and immunotherapy regimens have not been investigated in detail in larger cohorts. Accordingly, despite evidence in the existing literature highlighting inferior clinical outcomes in LO-NMOSD, the underlying factors driving these poor outcomes remain unclear. Consequently, the targets for improving outcomes in these patients are still unidentified.

The aim of the present study was to evaluate, within a sufficiently large European cohort of patients with NMOSD- where MOGAD has been reliably excluded- whether differences exist between LO- and EO-NMOSD in attack rates, manifestations, outcomes, and long-term disease burden at disease onset. This aims to identify key strategies for optimizing the care of patients affected by LO-NMOSD. For this purpose, we conducted a comprehensive analysis (“**C**haracterization **o**f prognosis, **t**herapy and **e**valuation of **r**esponse in **LO**-NMOSD [COPTER-LO Study]”) of the database of the Neuromyelitis Optica Study Group (NEMOS; www.nemos-net.de).

## Methods

### Study design and data collection

Patients were eligible if at least 18 years of age and meeting the diagnostic criteria for NMOSD with AQP4-IgG or for NMOSD without APQ4-IgG according to the 2015 criteria of the International Panel for NMO Diagnosis (IPND) (1). Patients with MOGAD were strictly excluded. As previously described, the serum antibodies to AQP4- and MOG-IgG were assessed in all patients using well-established cell-based assays (CBAs) (16, 17). All data entered in the database before 11^th^ of October 2022 were considered. In total, 446 patients from 35 German academic centers were included.

The following demographic and clinical data were retrieved from the NEMOS registry: age, gender, disease duration, year of onset and diagnosis, time to diagnosis, follow-up time, AQP4- and MOG-IgG serostatus, diagnostic criteria, ethnicity, clinical manifestation at disease onset and at follow-up examinations, number and type of attacks, detailed attack treatment, immunotherapy, EDSS, outcomes, and comorbidities. The current study combines current retrospective and (since 2016) prospective longitudinal data. If a history of NMOSD existed prior to database entry, these data were retrospectively collected using medical records. Since inclusion in the NEMOS database, a) annual examinations and/or b) additional examinations in the context of an attack were carried out prospectively by the participating centers. Disability was measured using the EDSS by a trained physician. Disease duration was defined as the time between onset and last documented visit. To assess disability accumulation during the attack, the relapse-associated worsening (RAW) was calculated defined as the difference between basal and late EDSS. Basal EDSS was the last EDSS before a definite attack, while late EDSS was the first EDSS measured at least 90 days after a definite attack, if no further event occurred in between. In case of an attack at disease onset the basal EDSS was set to 0. EDSS scores were only used for analysis of the longitudinal development of disability if collected during a clinically stable period and confirmed at subsequent study visit. The attack recovery analysis included only those attacks with available remission data. Complete remission without residuals was considered as full recovery. Having minor residuals after attack was considered as almost full remission. No improvement was rated as no recovery and incomplete remission as partial recovery (18). Immunotherapies were classified into B-cell-depletion (rituximab, inebilizumab), classical immunosuppressive treatment (azathiophrine, mycophenolate mofetil, methotrexate, oral steroids), interleukin-6-receptor-inhibition (satralizumab, tocilizumab), complement inhibition (eculizumab) and “others” (glatiramer acetate, interferon-beta, mitoxantrone, natalizumab, cyclophosphamide, alemtuzumab, dimethyl fumarate, intravenous immunoglobulins [IVIG]).

### Standard protocol approvals, registrations and patient consent

This study was part of the German Ministry of Education and Research (BMBF)-funded German Competence Network Multiple Sclerosis (KKNMS). The NEMOS cohort/KKNMS Nation^NMO^-Study was approved by the Medical Ethics Committee of Hannover Medical School (no*. 2009-5220*) and by all other participating centers. All patients provided written informed consent before enrollment.

### Statistical analysis

Statistical analysis was performed with SPSS version 27.0 (IBM Corp., Armonk, NY, USA) and figures were created with Prism version 8.4.3 (GraphPad Software, La Jolla, CA). Categorical values were presented as relative and absolute frequencies. Pearson`s chi-square test (χ2) or Fisher exact test was used to compare categorical data (differences in distribution of sex, antibody status, clinical manifestation, autoimmune and non-autoimmune comorbidities, immunotherapies and acute attack treatment). Continuous data was summarized with mean and standard deviation (SD) or median and range or interquartile range (IQR). Mann-Whitney U or Wilcoxon-test was used to compare non-parametric data (differences of age at onset and at diagnosis, differences in disease duration, absolute number or attacks, annualized attack rate, and EDSS). Annualized attack rate (AAR) was calculated by dividing the total number of attacks by the disease duration. Only patients with at least 12 months of follow-up time were included. To evaluate attack recovery, RAW was calculated between basal and late EDSS, as detailed above. The time from disease onset to reach EDSS ≥ 4, 6 and 8 was estimated through Kaplan-Meier estimator. The difference in rates of reaching EDSS 4, 6 and 8 were assessed using Cox proportional hazard regression analysis and adjusted for sex, antibody status, immunotherapy (yes/no) and year of disease onset. Survival curves between LO- and EO-NMOSD were compared using log-rank test.

Generalized estimating equations (GEE) were employed in an exploratory, univariate approach to analyze attack outcomes, accounting for the fact that patients could experience multiple relapses over time. This method allowed for the correlation between repeated measures from the same individual to be properly managed, providing robust estimates of the impact of onset type, treatment, and relapse symptoms on recovery. We assessed how the odds ratio (OR) for full recovery varied across different attack types and treatment modalities. Odds ratios were derived by exponentiating the regression coefficients (B-values). Confidence interval (CI) for ORs were similarly derived by exponentiating the 95% CI of the B-values. The autoregressive (AR[1]) correlation structure was applied, assuming stronger correlation in temporally close observations (19). A Generalized linear mixed model analysis (GLMM) was used to analyze full recovery as the outcome, adjusting for age at attack, sex, treatment type and diagnosis.

Frequency of a stable clinical course under therapy with rituximab, the most commonly used treatment for NMOSD in this cohort, was compared 6, 12, 36 and 60 months after the start of treatment. Post-treatment AAR was calculated as the number of attacks after cessation of long-term immunotherapy divided by the time between cessation of treatment and last follow-up.

## Results

### Characteristics of the NEMOS NMOSD cohort

This multicenter study included 446 NMOSD patients, 153 (34%) had a late-onset with an average age at onset of 59 years. The patients were predominantly female (83%) and of white descent (92%). The gender and ethnicity distributions did not differ significantly between LO- and EO-NMOSD (**Table 1**).

**Table 1:** Demographic characteristics, serostatus, attack type at disease onset and comorbidities categorized by age at disease onset^1^.

	Available n data	NMOSD (N=446)	LO-NMOSD (n=153)	EO-NMOSD (n=293)	*p*-value
Demography					
Female, n (%)	445	371 (83.4%)	123 (80.4%)	248 (84.9%)	0.222
Age at onset, median (range), y	446	43 (5-84)	59 (50-84)	34 (5-49)	**<0.001**
Age at diagnosis, median (range), y	446	47 (6-85)	61 (51-85)	39 (6-68)	**<0.001**
Age at database entry, median (range), y	317	52 (18-85)	65.0 (51-85)	45 (18-77)	**<0.001**
Time to diagnosis, median (range)^2^, y	443	1.0 (0-41)	0.0 (0-18)	2.0 (0-41)	**<0.001**
Follow-up time, median (range), y	345	1.8 (0-14.67)	1.3 (0-7)	1.9 (0-14.67)	0.085
Disease duration, median (range), y^3^	346	8 (0-52)	5 (0-27)	9 (0-52)	**<0.001**
Antibodies					
AQP4-IgG positive, n (%)	446	381(85.4%)	144 (94.1%)	237 (80.9%)	**<0.001**
AQP4-IgG and MOG-IgG negative, n (%)	446	65 (14.6%)	9 (5.9%)	56 (19.1%)	**<0.001**
AQP4-IgG positive female, n (%)	371	336 (90.6%)	117 (95.1%)	219 (88.3%)	**0.034**
AQP-4 and MOG-IgG negative female, n (%)	65	35 (9.4%)	6 (4.9%)	29 (11.7%)	
Ethnicity, n (%)	427				0.322
Whites		394 (92%)	137 (95%)	257 (91%)	
Asian		7 (1.6%)	2 (1%)	5 (2%)	
Arabic		10 (2%)	1 (1%)	9 (3%)	
Latin		4 (1%)	2 (1%)	2 (1%)	
African		9 (2%)	2 (1%)	7 (3%)	
Other		3 (1%)	0	4 (1%)	
Attack type at disease onset, n (%)					
Optic neuritis	428	160 (37.4%)	40 (27.4%)	120 (42.6%)	**0.002**
Myelitis	428	192 (44.9%)	85 (58.4%)	107 (37.9%)	**<0.001**
Optic neuritis and myelitis	428	18 (4.2%)	5 (3.4%)	13 (4.6%)	0.677
Brainstem encephalitis	428	10 (2.3%)	1 (0.7%)	9 (3.2%)	0.056
Area postrema syndrome	428	9 (2.1%)	2 (1.4%)	7 (2.5%)	0.589
Diencephalic syndrome	428	NA	NA	NA	NA
Cerebral syndrome	428	2 (0.5%)	1 (0.7%)	1(0.4%)	0.631
Multiple symptoms	428	18 (4.2%)	6 (4.1%)	12 (4.4%)	0.823
Other	428	19 (4.4%)	6(4.1%)	13 (4.6%)	0.834
Comorbidities, n (%)					
Autoimmune comorbidities^4^	412	134 (32.5%)	42 (30.4%)	92 (33.6%)	0.521
Hashimoto thyroiditis		40 (29.9%)	12 (28.6%)	28 (30.4%)	
SLE		34 (25.4%)	7 (16.7%)	27 (29.3%)	
Sjögren´s syndrome		20 (14.9%)	5 (11.9%)	15 (16.3%)	
Myasthenia gravis		12 (9.0%)	4 (9.5%)	8 (8.7%)	
Rheumatoid arthritis		8 (6.0%)	7 (16.7%)	1 (1.1%)	
Other ^5^		64 (47.8%)	31 (73.8%)	33 (35.9%)	
Non autoimmune comorbidities	404	233 (57.7%)	101 (71.2%)	132 (50.2%)	**<0.001**
Cardiovascular diseases	343	87 (21.5%)	56 (40.0%)	31 (11.7%)	**<0.001**
Oncological diseases	343	37 (9.2%)	25 (17.9%)	12 (4.6)	**<0.001**

^1^Percentages may not add exactly to 100% because of rounding. Bold values indicate statistically significant results. ^2^Time between onset and NMOSD diagnosis in years. ^3^Time between onset and last follow up. ^4^Each autoimmune comorbidity was considered individually.^5^Other: Type 1 diabetes mellitus, psoriasis, autoimmune hepatitis, vitiligo, ankylosing spondylitis, Crohn´s disease, Grave´s disease, celiac disease, idiopathic thrombocytopenic purpura, uveitis, iritis, primary biliary cirrhosis, scleroderma.

AQP4-IgG, aquaporin-4 immunoglobulin G; MOG-IgG, myelin oligodendrocyte glycoprotein immunoglobulin G; NMOSD, Neuromyelitis optica spectrum disorders; LO, late-onset; EO, early-onset; y, years; n/a, not available, SLE, Systemic Lupus Erythematosus; NA, not available.

The majority (85.4%, n = 381) of the NEMOS study cohort met the IPND criteria for AQP4-IgG positive NMOSD, with a higher frequency of AQP4-IgG positive patients observed in LO- than in EO-NMOSD (94.1% vs. 80.9%, *p* < 0.001). The proportion of females in the AQP4-IgG positive subgroup was higher in LO-NMOSD patients (95.1% vs. 88.3%, *p* < 0.034).

The median disease duration of the whole cohort was 8 years (range 0 - 52) and differed between LO- and EO-NMOSD patients (5 vs. 9 years, *p* < 0.001). LO-NMOSD patients showed a shorter time from onset to diagnosis (0 vs. 2 years, *p* < 0.001).

Comorbidities were observed in both groups: As expected, LO-NMOSD patients were affected more often by non-autoimmune comorbidities than EO-NMOSD patients (71.2% vs. 50.2%, *p* < 0.001). There was no difference in the number of concomitant autoimmune diseases in general (30.4% vs. 33.6%, *p* = 0.521).

### Disease onset

The most common manifestation at first attack were myelitis (n=192/428, 44.9%) and optic neuritis (n = 160/428, 37.4%, **Table 1**). The frequency of combined optic neuritis and myelitis at onset was low (n = 18/428, 4.2%). Other core clinical characteristics of NMOSD were rare or absent at first attack (area postrema syndrome [n = 9/428, 2.1%], other acute brainstem syndrome [n = 10/428, 2.3%], cerebral symptoms [n = 2/428, 0.5%], or diencephalic syndrome [n = 0/428, 0%]). Optic neuritis as an initial manifestation was observed more frequently in EO-NMOSD patients (42.6% vs. 27.4%, *p* < 0.002), while transverse myelitis was more common in LO-NMOSD patients (58.4% vs. 37.9%, *p* < 0.001). The remaining primary manifestations showed no difference between LO and EO-NMOSD. Brainstem encephalitis (n = 10 in the whole cohort) occurred numerically more often in EO-NMOSD patients, although this was not statistically significant. (3.2% vs. 0.7%, *p* = 0.056).

### Attack data

To compare the frequency of attacks between LO- and EO-NMOSD, subgroups of different attack types were formed (**Table 2**). The calculated AAR showed no difference between LO- and EO-NMOSD regarding all attacks (0.51 vs. 0.54, *p* = 0.352), myelitis (0.37 vs. 0.35 *p* = 0.275), optic neuritis (0.35 vs. 0.25, *p* = 0.690) and combined attack of optic neuritis and myelitis (0.15 vs. 0.11, *p* = 0.240).

**Table 2:** Detailed attack data of NMOSD patients, categorized by age at disease onset.

	Available n	NMOSD	LO-NMOSD	EO-NMOSD	*p*-value^1^
ARR, mean (SD)^2^	211				
Total attacks		0.53 (0.44)	0.51 (0.51)	0.54 (0.40)	0.352
Myelitis attacks		0.36 (0.31)	0.37 (0.28)	0.35 (0.31)	0.275
Optic neuritis attacks		0.27 (0.36)	0.35 (0.62)	0.25 (0.26)	0.690
Optic neuritis and myelitis attacks		0.11 (0.07)	0.15 (0.10)	0.11 (0.06)	0.240
Monophasic course, n (%)	340	27 (7.9%)	13 (12.3%)	14 (6.0%)	**0.047**
Time to second attack, months (median, range)	310	11.5 (1-491)	8 (1-220)	12.5 (1-491)	0.104
RAW^3^, median (IQR)					
RAW at all clinical attacks^4^	184	1.5 (0-3)	3 (1.5-6)	0.5 (0-2)	**<0.001**
RAW^5^ at onset	94	3.0 (2-5)	4.0 (2.5-6.5)	2.5 (1.5-3)	**<0.001**
IVMP therapy^6^, n (%)	1254^6^				
IVMP alone		1014 (80.9%)	208 (72.2%)	806 (83.4%)	**<0.001**
IVMP with PE/IA		240 (19.1%)	80 (27.8%)	160 (16.5%)	**<0.001**
Total dose of IVMP mg/attack, mean (SD)	775	5192 (3145)	6052 (3563)	4961 (2984)	**<0.001**
Apheresis therapy, n (%)	338				
Plasma exchange		258 (76.3%)	70 (72.9%)	188 (77.7%)	0.505
Immunoadsorption		53 (15.6%)	18 (18.8%)	35 (14.5%)	0.505
Plasma exchange + immunoadsorption		27 (7.9%)	8 (8.3%)	19 (7.8%)	0.505
Apheresis therapy cycles, mean (SD)	287	6.75 (2.5)	6.66 (2.46)	6.79 (2.52)	0.592

^1^Bold values indicate statistically significant results. ^2^Annualized attack rate (Number of total attacks divided by disease duration), symptom specific stratification. Only patients with at least 12 month of follow-up time were included. ^3^RAW: EDSS difference between basal EDSS before attack and EDSS ≥ 90 days after attack, if no further attack occurred. ^4^Median basal EDSS before attack: EO-NMOSD = 3.5 and LO-NMOSD = 3.0. ^5^RAW after disease onset.^6^Intravenous methylprednisolone and apheresis therapy (plasma exchange and/or immunoadsorption), subgroup specific for late- and early-onset. ^6^1254 attacks from 199 (67.9%) EO-NMOSD and 107 (69.9%) LO-NMOSD patients. Due to missing data, detailed analysis of 200 attacks was not possible.

NMOSD, Neuromyelitis optica spectrum disorder; LO, late-onset; EO, early-onset; EDSS, Expanded Disability Status Scale; ARR, Annualized Attack Rate; PE, Plasmaexchange; IA,Immunoabsorption; IVMP, Intravenous methylprednisolone pulse; IQR, interquartile range; SD, standard deviation; mg, milligram; RAW, relapse associated worsening.

The time from onset to second attack showed no differences between LO- and EO-NMOSD (8 vs. 12.5 months, *p* = 0.104), with a considerably wider time span for EO-NMOSD of almost 41 years (**Table 2**). A monophasic disease course was observed in 12.3% of patients with late onset and in 6.0% of patients with early onset (*p* = 0.047).

### Recovery after attack

**Figure 1:**
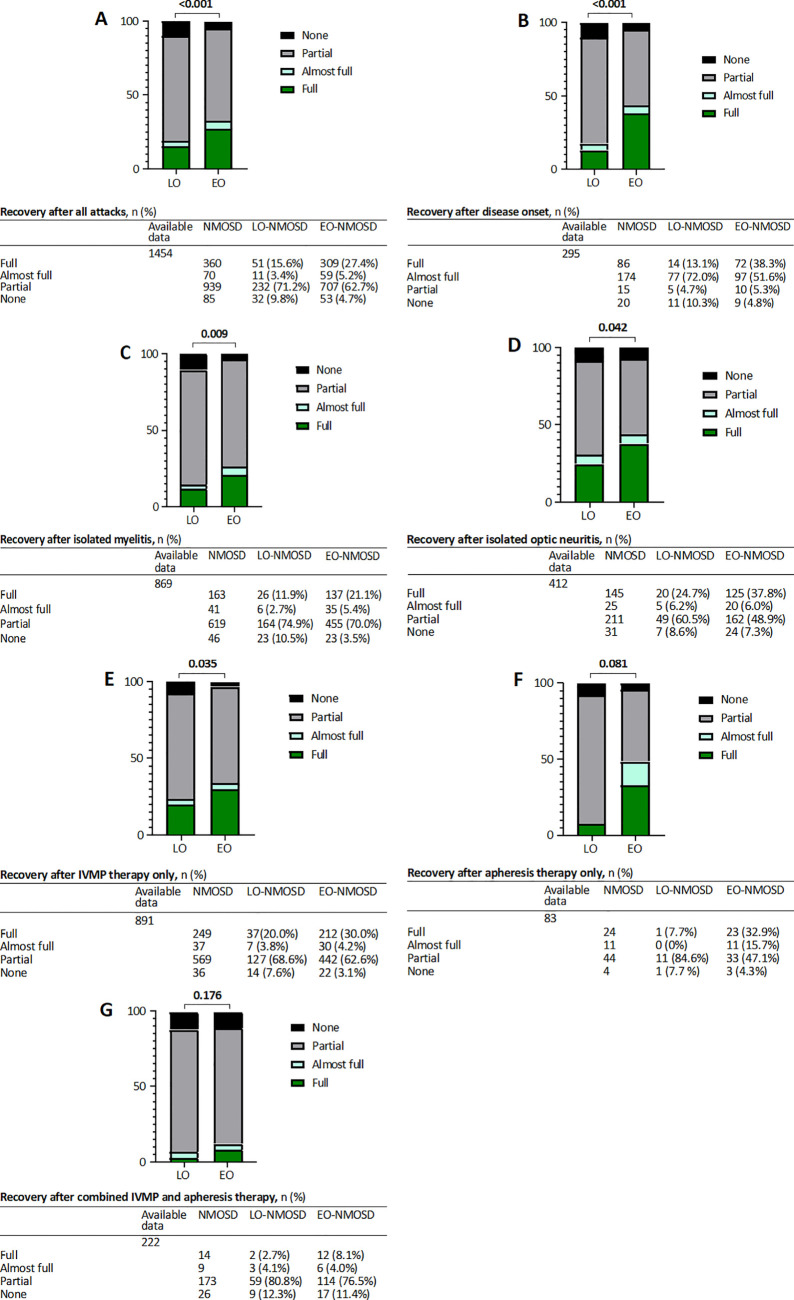
Recovery data after attack and attack treatment. Shown are the percentage of attack recovery after all attacks **(A)**, attack at onset **(B)**, isolated myelitis **(C)**, isolated optic neuritis **(D)**, IVMP therapy only **(E)**, apheresis therapy only **(F)** and after combined IVMP and apheresis therapy **(G)**. Percentages may not add exactly to 100% because of rounding.

We analyzed 1454 attacks with remission data from 353 NMOSD patients (EO-NMOSD: n = 1128, LO-NMOSD: n = 326). Complete recovery was achieved in every fourth attack (n= 360/1454 24.8%) in the entire study population. Irrespective of the applied treatment regimen, EO-NMOSD patients had a better recovery rate than LO-NMOSD patients (**Figure 1A**): Only 15.6% of LO-NMOSD patients achieved complete recovery compared to 27.4% of EO patients. Furthermore, 9.8% of LO-NMOSD patients did not experience any recovery compared to 4.7% of EO-NMOSD patients. GEE analysis with an odds ratio of 0.446 (95% CI 0.297 – 0.732, *p* < 0.001) indicate that LO-NMOSD patients have relevant lower odds of achieving full recovery compared to EO-NMOSD patients (**Table 3**). Moreover, a comparison of complete recovery fractions of subgroups with isolated optic neuritis and myelitis was conducted. Of the 869 isolated attacks of myelitis analyzed (**Figure 1C**), the differences in recovery between LO- and EO-NMOSD were observed for both complete (11.9% vs 21.1%) and no recovery (10.5% vs 3.5%). In contrast 412 analyzed optic neuritis attacks differed only in the frequency of complete remission (24.7% vs. 37.8%, **Figure 1D**). LO-NMOSD patients with isolated myelitis or optic neuritis attacks have lower odds of achieving full recovery compared to patients with EO-NMOSD (myelitis: OR = 0.481 [95% CI 0.277 – 0.834], *p* = 0.009; optic neuritis: OR= 0.470 [95% CI 0.327 – 0.972], *p* = 0.042). Furthermore, 295 patients with outcome data for the attack at disease onset were evaluated. Complete remission was reached in 13.1% of LO-NMOSD patients compared to 38.3% of EO-NMOSD patients (**Figure 1B**). Therefore, LO-NMOSD patients demonstrated lower odds of achieving full recovery at disease onset compared to EO-NMOSD patients (OR: 0.244 [95% CI 0.129 – 0.460], *p* < 0.001). Similarly, the GLMM analysis showed that older age at attack was significantly associated with a lower likelihood of full recovery across all attacks, as well as in the isolated myelitis and isolated optic neuritis subgroup (**Supplementary Table 6**). Moreover, full recovery fractions decreased with age from 27.4% in early-onset to 16.7% in late-onset and 6.3% in very late- onset (onset age ≥ 75 years). Additionally, LO-NMOSD patients showed a higher RAW after attack than EO patients, irrespective of whether all attacks are considered (Δ3 vs. Δ0.5, *p <*0.001, **Table 2**) or only the attack at disease onset (Δ4 vs. Δ2.5, *p <*0.001, **Table 2**).

**Table 3:** Odds ratios for full recovery based on different attack types and treatment modalities between LO- and EO-NMOSD patients in generalized estimating equations (GEE) analysis.

	OR (95% CI)	*p-*value^1^
Recovery		
**All attacks**	0.446 (0.279- 0.732)	**<0.001**
**Disease onset**	0.244 (0.129 – 0.460)	**<0.001**
**Isolated Myelitis**	0.481 (0.277 – 0.834)	**0.009**
**Isolated optic neuritis**	0.470 (0.327– 0.972)	**0.042**
**IVMP therapy only**	0.555 (0.321 – 0.670)	**0.035**
**Apheresis therapy only**	0.153 (0.019 – 1.264)	0.081
**Combined IVMP and apheresis therapy**	0.335 (0.104 - 1.578)	0.176

^1^Bold values indicate statistically significant results.

CI, confidence interval; EDSS, Expanded Disability Status Scale; EO, early onset; GEE, Generalized Estimating Equation; IVMP, intravenous methylprednisolone; LO, late onset; OR, Odds Ratio; NMOSD, Neuromyelitis optica spectrum disorder.

### Attack treatment

We reviewed 1254 attacks, of which 80.9% were treated with high dose intravenous methylprednisolone (IVMP) only and 19.1% with steroid and plasmapheresis or immunoadsorption (**Table 2**). Due to missing data, detailed analysis of 200 attacks was not possible. The proportion of patients who received both IVMP and apheresis therapy was higher in LO- than in EO-NMOSD (27.7% vs. 16.5%, *p* < 0.001, **Table 2**). Moreover, mean dose of steroids per attack was higher in LO-NMOSD patients (6052 mg [± SD 3563], n = 164 attacks; vs. 4961 mg [± SD 2984], n = 611 attacks; *p* < 0.001, **Table 2**).

Among all analyzed attacks treated with steroids only, 891 attacks were documented with corresponding data on attack remission, 185 from LO- und 706 from EO-NMOSD patients. The proportion of fully recovered attacks after steroid treatment was higher in EO-NMOSD patients (complete: 20.0% vs. 30.0%, **Figure 1E**) and LO-NMOSD patients have reduced odds of achieving complete recovery after IVMP therapy (OR: 0.555 [95% CI 0.321 – 0.670], *p* = 0.035). Of 83 attacks treated exclusively with apheresis therapy (**Figure 1F**), only 7.7% of LO-NMOSD patients recovered completely compared to 32.9% of patients with EO (OR: 0.153 [95% CI 0.019 - 1.264, *p* = 0.081]). The combined treatment with steroids and apheresis therapy (n = 222, **Figure 1G**) showed no significant differences in recovery fractions between LO- and EO-NMOSD (OR 0.335 [95% CI 0.104- 1.578], *p* = 0.176).

### Course of the disease

**Figure 2:**
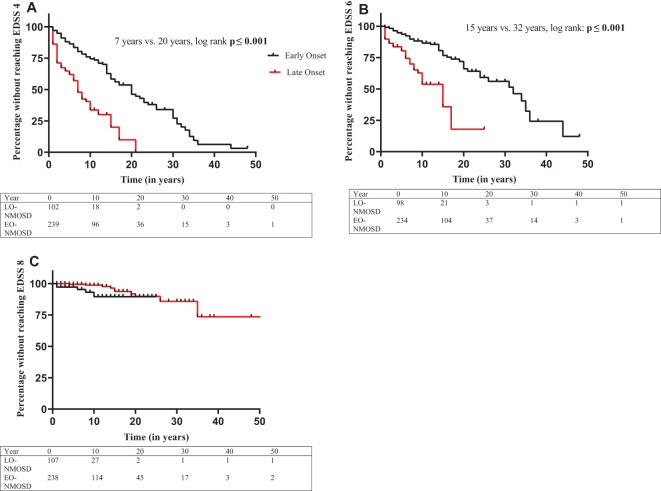
Time from disease onset to EDSS ≥4, 6 and 8, categorized by age at disease onset. Years from disease onset to EDSS ≥4 **(A)**, 6 **(B)**, 8 **(C)**. All three Kaplan-meier survival curves demonstrate significant faster EDSS progression in LO-NMOSD. Disability values were included if collected at regular follow-up, attack-independent and confirmed in a subsequent visit (C: medians could not be calculated, log rank *p*=0.016).

LO-NMOSD patients had a higher risk of reaching EDSS scores above 4, 6 or 8 than EO-NMOSD patients (**Figures 2A-C**). The median time from onset to an EDSS ≥ 4 was 7 years in LO- and 20 years in EO-NMOSD patients (*p* < 0.001), the time to an EDSS ≥ 6 was 15 years in LO- and 32 years in EO-NMOSD patients (*p* < 0.001). No median values for the time to reach an EDSS ≥ 8 could be calculated due to small amount of data, but Kaplan-Meier curves showed differences between LO- and EO-NMOSD (*p* = 0.002). In the adjusted cox proportional hazard regression analysis (**Table 4**), the risk of reaching EDSS 4, 6 and 8 was higher among patients with LO-NMOSD compared with EO-NMOSD (EDSS 4: aHR 2.64 [95% CI 1.81 - 3.84]; EDSS 6: aHR 3.25 [95% CI 1.95 - 5.43]; EDSS 8: 3.52 [95% CI 1.44 - 8.59]).

**Table 4:** Risk of reaching disability milestones from disease onset in LO-NMOSD compared to EO-NMOSD.

	HR (95% CI)	Adjusted HR^1^ (95% CI)
From disease onset to		
**EDSS 4**	3.54 (2.47-5.10)	2.64 (1.81-3.84)
**EDSS 6**	3.98 (2.47-6.43)	3.25 (1.95-5.43)
**EDSS 8**	5.79 (2.48-13.510)	3.52 (1.44-8.59)

^1^Adjusted for sex, year of NMOSD disease onset, antibody status, immunotherapy (yes/no).

CI, confidence interval; EDSS, Expanded Disability Status Scale; EO, early onset; HR, Hazard Ratio; LO, late onset; NMOSD, Neuromyelitis optica spectrum disorder.

### Long-term immunotherapy

Long-term immunotherapy was documented in 90.4% (n = 403/446) of all NMOSD patients. LO-NMOSD patients had a shorter time from onset to initiation of long-term treatment than EO-NMOSD patients (6.0 vs. 15.0 months, *p* = 0.030, **Table 5**).

**Table 5:** Detailed data on long-term immunotherapies, categorized by age at disease onset.

	Available n	NMOSD	LO-NMOSD	EO-NMOSD	p-value^1^
Immunotherapy					
Immunotherapy (yes vs. no/unknown)	446	403 (90.4%)	134 (87.6%)	269 (91.8%)	0.229
Switch in immunotherapy (yes vs. no/unknown)	380	176 (47.3%)	53 (41.7%)	123 (48.6%)	0.204
Time from onset to immunotherapy, months (median, range)	358	10 (0-562)	6 (0-210)	15 (0-562)	**0.030**
**First line immunotherapy^2^**	394				
B-cell depletion		172 (43.7%)	63 (48.1%)	109 (41.4%)	0.210
Classical immunosuppressants		132 (33.5%)	43 (32.8%)	89 (33.8%)	0.840
IL-6-receptor inhibition		2 (0.5%)	0 (0%)	2 (0.8%)	0.317
Complement inhibition		14 (3.6%)	9 (6.9%)	5 (1.9%)	**0.019**
Other		65 (16.5%)	11 (8.4%)	54 (20.5%)	**0.002**
**Immunotherapy used at the last follow-up**	390				
B-cell depletion		241 (61.8%)	84 (63.6%)	157 (60.9%)	0.592
Classical immunosuppressants		83 (21.3%)	26 (19.7%)	57 (22.1%)	0.584
IL-6-receptor inhibition		34 (8.7%)	9 (6.8%)	25 (9.7%)	0.341
Complement inhibition		24 (6.2%)	13 (9.8%)	11 (4.3%)	**0.030**
Other		8 (2.1%)	0 (%)	8 (3.1%)	0.056
Post-treatment ARR^3^ (mean, SD)	207	0.30 (0.51)	0.23 (0.45)	0.32 (0.53)	**0.026**
Attack-free under RTX first-line^4^					
Attack-free after 6 mo. n, (%)	128	100 (78.1%)	35 (77.8%)	65 (78.3%)	0.944
Attack free after 12 mo. n, (%)	126	92 (73.0%)	34 (75.6%)	58 (71.6%)	0.632
Attack free after 36 mo. n, (%)	86	49 (57.0%)	17 (56.7%)	32 (57.1%)	0.966
Attack free after 60 mo. n, (%)	61	25 (41.0%)	9 (42.9%)	16 (40.0%)	0.829
Attack-free under RTX any line^5^					
Attack free after 6 mo. n, (%)	194	158 (81.4%)	54 (84.4%)	104 (80.0%)	0.461
Attack free after 12 mo. n, (%)	185	143 (77.3%)	53 (84.1%)	90 (73.8%)	0.111
Attack free after 36 mo. n, (%)	135	83 (61.5%)	29 (64.4%)	54 (60.0%)	0.617
Attack free after 60 mo. n, (%)	99	46 (46.5%)	14 (45.2%)	32 (47.1%)	0.861

NMOSD, Neuromyelitis optica spectrum disorder; LO, late-onset; EO, early-onset; RTX, rituximab; AZA, azathioprine; MTX, metothrexate; MMF, mycophenolate mofetil; IL-6, interleukin-6; ARR, annualized attack rate.

^1^Bold values indicate statistically significant results.

^2^Immunotherapy: B-cell depletion (RTX/inebilizumab), classical immunosuppressants (AZA, MMF, MTX, oral steroids), IL6-receptor inhibition (tocilizumab/satralizumab), Complement inhibition (eculizumab), Other (glatiramer acetate, interferon beta, mitoxantrone, fingolimod, alemtuzumab, natalizumab, dimethyl fumarate, intravenous immunoglobulins, cyclophosphamide).

^3^Post treatment ARR = Number of attacks after initiation of NMOSD therapy divided by the time between initiation and last follow-up.

^4^Frequency of clinical stable course after 6, 12, 36, and 60 month after first line therapy with RTX.

^5^Frequency of clinical stable course after 6, 12, 36, and 60 month after treatment during the course of the disease with RTX.

Nearly half of the patients (n = 63/131, 48.1%) of LO-NMOSD received B-cell depleting immunotherapy with rituximab or inebilizumab as first-line treatment compared to 41.4% (n= 109/263) of EO-NMOSD patients (*p* = 0.210, **Table 5**). Classical immunosuppressants (azathioprine, methotrexate, mycophenolate-mofetil and oral corticosteroids) were used in 32.8% (n = 43/131) of LO- and 33.8% (n = 89/263) of EO-NMOSD patients as first-line therapies (*p* = 0.840, **Table 5**). Interleukin-6 receptor inhibition (tocilizumab/satralizumab: 0% [n = 0/131] vs. 0.8% [n = 2/263], *p* = 0.317, **Table 5**) and complement inhibition (eculizumab: 6.9% [n = 9/131] vs. 1.9% [n = 5/263], *p* = 0.019, **Table 5**) were less frequently used in EO- and LO-NMOSD. Other immunotherapies, mostly treatments approved for multiple sclerosis (MS) in those who were initially misdiagnosed, were more commonly used in EO-NMOSD (8.4% [n = 11/131] vs. 20.5% [n = 54/263] *p* = 0.002).

At last follow-up the fraction of NMOSD patients receiving B-cell depleting immunotherapy had increased, with no difference between the LO- and EO-NMOSD group (63.6% [n = 84/132] vs. 60.9% [n = 157/258], *p* = 0.592). The proportion of Interleukin-6 receptor inhibition (6.8% [n = 9/132] vs. 9.7% [n = 25/258], *p* = 0.341) and complement inhibition (9.8% [n = 13/132] vs. 4.3% [n = 11/258], *p* = 0.030) increased in both LO- and EO-NMOSD. Classical immunosuppressants were used in 19.7% (n = 26/132) of LO- and 22.1% (n= 57/258) of EO-NMOSD patients at last follow-up (*p* = 0.584). Other immunotherapies decreased in LO- (0%, n = 0/132) and EO-NMOSD (3.1% [8/258]).

Among the most commonly used long-term immunotherapy, rituximab, there was no difference in the proportion of LO- and EO-NMOSD patients who remained attack-free 6, 12, 36, and 60 months after starting therapy irrespective of whether rituximab was used after initial diagnosis or later in the disease course (**Table 5**). Both groups experienced a similar remarkable reduction of attacks after initiation of NMOSD-specific long-term immunotherapy (**Table 5**).

### Differences between AQP4-IgG positive and negative LO-NMOSD patients

To determine whether the AQP4-IgG status introduces bias in our results, we performed a subgroup analysis of AQP4-IgG-negative NMOSD patients (**Supplementary Tables 2.1-5.2**). The core findings of our analyses were confirmed within this subgroup. Accordingly, in the late-onset group, seronegative patients exhibited a higher prevalence of myelitis (66.7% vs. 31.5%, *p* = 0.042) as the initial manifestation, similar to the seropositive group (57.7% vs. 39.5%, *p* = 0.001). There was no difference in the AAR (AQP4-IgG negative: 0.46 vs. 0.54, *p* = 0.799; AQP4-IgG positive: 0.51 vs. 0.54, *p* = 0.292) and both late-onset groups demonstrated lower odds of achieving full recovery after an attack (AQP4-IgG negative: OR 0.241 [95% CI 0.065- 0.899], *p* = 0.034; AQP4-IgG positive: OR 0.465 [95% CI 0.298 - 0.728], *p* < 0.001). Notable findings not observed in the seropositive group include the absence of a difference in optic neuritis as the initial manifestation (11.1% vs. 44.4%, *p* = 0.072), as well as no difference in the prevalence of monophasic disease courses (0% vs. 7.1%, *p* = 0.466). The proportion of LO-NMOSD patients receiving long-term immunotherapy was comparable between groups (AQP4-IgG negative: 88.9% vs. 91.1%; *p* = 0.843; AQP4-IgG positive: 87.5% vs. 92.0%, *p* = 0.214). A higher percentage of AQP4-IgG-negative patients were treated with B-cell-depleting immunotherapy (AQP4-IgG negative: 87.5% vs. 38.8%, *p* = 0.018; AQP4-IgG positive: 45.5% vs. 42.1%, *p* = 0.536), while a greater fraction of AQP4-IgG-positive patients received therapies targeting newly approved mechanisms (**Supplementary Tables 5.1, 5.2**).

## Discussion

The occurrence of LO-NMOSD poses significant challenges, as prior research suggests a more severe disease course and poorer prognosis (**Supplementary Table 1**). However, some of these data came from relatively small cohorts or from cohorts that may have included patients with MOGAD.

Our study presents the first comprehensive analysis of disease-relevant factors in a large European cohort of NMOSD patients, with reliable exclusion of MOG-IgG positive patients.

We aimed to identify disease-related parameters that contribute to the worse outcome observed in LO-NMOSD patients. The NEMOS cohort of 153 LO-NMOSD and 293 EO-NMOSD patients is one of the largest investigated cohorts so far.

The findings confirm a difference in initial manifestation between LO- and EO-NMOSD. Particularly, myelitis at disease onset was the most common manifestation in LO-NMOSD and occurred more frequently than in EO-NMOSD, while optic neuritis was more prevalent in EO-NMOSD patients. These results are in line with previous studies concerning NMOSD and align with other studies, which similarly identified age-related differences in MS and MOGAD (8, 20–23). A plausible explanation for the higher prevalence of myelitis in LO-NMOSD may be the notion of an increased vulnerability of the spinal blood barrier (8).

Prior studies have suggested differences in accumulation of disability between LO- and EO-NMOSD (8, 15). Our analysis further revealed that the severity of attacks and limited remission, particularly from myelitis attacks, significantly contribute to the poorer outcome observed in LO-NMOSD patients. Notably, the AAR were comparable between LO- and EO-NMOSD. Consistent with previous research, we also observed a faster progression from onset to disability milestones in LO-NMOSD (7, 8, 15, 24, 25). Furthermore, LO-NMOSD patients were found to be at least two to three times more likely to reach EDSS milestones than EO-NMOSD patients, comparable to the adjusted hazard ratio observed in late-onset MS patients (23). Many LO-NMOSD patients, especially those with myelitis at onset, do not fully recover, start at a higher EDSS and therefore reach higher disability scores faster than EO-NMOSD patients (26, 27).

Our cohort also demonstrated poorer outcomes from optic neuritis attacks in LO-NMOSD. Similarly, a previous study found that LO-NMOSD patients have worse visual acuity at onset and poorer long-term visual outcomes compared to EO-NMOSD (13). A comparison between pediatric and adult patients revealed that visual recovery after optic neuritis was poorer in the adult group with MOGAD (28). This has been suggested to reflect an age-dependent decline in neuroplasticity of the visual system at the cortical level (28).

With increasing age, repair mechanisms may decline (5), reducing the capacity for phagocytosis and motility (29–32). Additionally, age-related decrease in vascular supply might impair spinal cord recovery (33), with LO-NMOSD patients who have cardiovascular comorbidities potentially facing a greater risk of poorer recovery. Future studies should investigate the impact of comorbidities on the disease course and recovery in NMOSD and explore age-related differences within the LO-NNMOSD group.

Moreover, in the field of MS age and disease duration have been identified as important limiting endogenous factors for remyelination (34, 35). Cellular aging and the loss of regeneration potential in neuronal and glial cells may contribute to poorer outcomes in late-onset neuroimmunological diseases (23, 36). Currently, there is a lack of NMOSD-specific models to explore the effects of aging on demyelination and remyelination.

In contrast to other studies, we have obtained additional detailed information on attack treatment regimens. In this cohort, higher doses of steroids were administered to LO-NMOSD patients during attacks than to EO-NMOSD, and a larger proportion received apheresis therapy. Other studies did not identify differences in the frequency of apheresis therapy as acute attack treatment (8, 14), but differences in treatment regimens between countries and centers may play a role as well as the lower number of attacks analyzed in those studies. One potential explanation may be the focus on the absolute number of patients in these studies, in comparison to the number of attacks in this work. As LO-NMOSD patients received more intensive acute attack therapy compared to EO-NMOSD patients, out data do not indicate that the use of acute therapy itself is responsible for the poorer outcome observed in LO-NMOSD patients. The worse outcome might be explained by the diminishing anti-inflammatory effects of steroids with increasing age (37, 38). Furthermore, it has been reported that increasing age is associated with a lower probability of achieving complete remission following apheresis therapy (39).

In this study, the proportion of patients receiving long-term immunotherapy was comparable between LO- and EO-NMOSD, with B-cell depleting therapies being the most frequently used. Other studies reported either significantly lower, similar, or no differences in general immunotherapeutic treatment regimens between LO- and EO-NMOSD (8, 9, 15, 25). The effect of B-cell directed therapies was comparable in both groups. Complement inhibition was used more frequently in LO-NMOSD patients, particularly as first-line treatment, and may have influenced the lower post-treatment AAR. The use of highly potent immunotherapy in the late-onset group may be based on the severity of the attacks. Further research is needed to investigate the efficacy of newly approved immunotherapies in LO-NMOSD patients. Of note, patients with LO-NMOSD were diagnosed sooner and long-term immunotherapy was initiated earlier than patients with EO-NMOSD in our cohort (9, 40). This could be attributed to the severity of the attacks and the higher proportion of AQP4-IgG positive patients in LO-NMOSD patients. Previous studies have reported contradictory results. Some found no difference in time to diagnosis or initiation of immunotherapy between LO- and EO-NMOSD, despite differing disease severities. These inconsistencies may reflect methodological limitations or accessibility of AQP4-IgG CBA, potentially delaying diagnosis (9, 40, 41).

All in all this study demonstrated that disease progression in LO-NMOSD patients is mainly driven by suboptimal recovery from attacks, rather than delays in diagnosis or long-term immunotherapy. This highlights the need for new acute attack strategies in LO-NMOSD patients to optimize attack outcome. As an antibody-mediated disease, AQP4-IgG positive NMOSD may benefit from add-on therapies targeting the neonatal Fc receptor (FcRn). FcRn maintains high IgG autoantibody levels in serum (42, 43). Blocking FcRn promotes the degradation of IgG autoantibodies, thereby reducing pathogenic autoantibodies (44). Phase 3 trials have shown that it can reduce serum IgG levels within one week, supporting its potential in autoimmune neurological diseases (45). The first studies have investigated FcRn antagonists as an add-on therapy for acute NMOSD attacks and showed improvements in EDSS and a reduction in AQP4-IgG and serum IgG levels among patients with LO-NMOSD (46, 47). However, results on clinical efficacy are limited by the small sample size and should be confirmed in larger controlled studies with a special focus on LO-NMODS patients. Another approach for AQP4-IgG positive NMOSD might be targeting the complement cascade or Interleukin-6 receptor pathway. Terminal complement inhibition rapidly suppresses AQP4-IgG-induced, complement-dependent inflammation and may reduce attack severity and further disability. This treatment is used in both acute and chronic settings for atypical hemolytic uremic syndrome, underlining its rapid complement activity suppression (48). Furthermore, several case reports have shown its rapid efficacy in myasthenia gravis (49, 50). To date, only two case reports have documented the use of complement inhibition as a third-line therapy for acute attacks in NMOSD, specifically in cases where IVMP and apheresis were ineffective (51, 52). Similarly, high-dose IVMP combined with Interleukin-6-receptor blockade has shown improvements in disability in NMOSD patients with myelitis (53). Further controlled studies are urgently needed to evaluate the impact of these novel therapeutic approaches on acute attack treatment in LO-NMOSD patients. For the heterogeneous group of antibody-negative NMOSD, developing effective therapeutic strategies is of great importance. However, due to the unclear pathophysiology, this remains a major challenge. Therefore, a precise characterization of this disease spectrum is essential to identify new therapeutic targets.

This study has certain limitations, including its partially retrospective design, partially incomplete EDSS and recovery data. Additionally, another key limitation is the lack of precise time records on the initiation of acute attack treatments. Furthermore, inconsistent data collection methods and difficulties in recalling past events or comorbidities may cause recall bias. Despite these limitations, the strengths of the study lie in its large patient cohort, reliable antibody status, extensive attack data, and its multicenter longitudinal design, which provides robust data over time in the context of a rare disease. Moreover, the proportion of missing attack data in relation to the total cohort was comparable between LO- and EO-NMOSD. A further strength of our study was the sub-analysis of AQP4-IgG-positive and negative LO-NMOSD patients.

## Conclusion

This study highlights that LO-NMOSD patients experience a severe disease course characterized by an increased incidence of myelitis at disease onset, worse attack outcomes and faster disability accumulation compared to EO-NMOSD patients. Rapid disability progression due to worse attack outcomes despite aggressive treatment of acute attacks and the early initiation of long-term immunotherapies highlights the need for novel therapeutic strategies to manage acute attacks and promote remyelination in LO-NMOSD.

## Data Availability

The raw data supporting the conclusions of this article will be made available by the authors, without undue reservation.
