# Supplementary material for: Worse recovery from acute attacks and faster disability accumulation highlights the unmet need for improved treatment in patients with late-onset neuromyelitis optica spectrum disorders (COPTER-LO study)
Source: Front Immunol. 2025 Apr 11;16:1575613. doi: 10.3389/fimmu.2025.1575613 (PMC12021610; doi:10.3389/fimmu.2025.1575613)
Supplement: Supplementary file 1 [file DataSheet1.pdf]

Supplement

Worse recovery from acute attacks and faster disability accumulation highlights the unmet need for improved treatment in patients with late-onset Neuromyelitis Optica spectrum disorder (NMOSD)

Julian Reza Kretschmer, Daria Tkachenko, Tania Kümpfel, Joachim Havla, Daniel Engels, Friedemann Paul, Patrick Schindler, Judith Bellmann-Strobl, Achim Berthele, Katrin Giglhuber, Clarissa Zappe, Luisa Klotz, Lisa Revie, Eva Dawin, Makbule Senel, Hayrettin Tumani, Florian Then Berg, Clemens Warnke, Markus Kraemer, Annette Walter, Antonios Bayas, Uwe K. Zettl, Ann-Sophie Lauenstein, Yavor Yalachkov, Thorleif Etgen, Matthias Kaste, Felix Luessi, Stefan Gingele, Sarah Passoke, Martin S. Weber, Jörn Peter Sieb, Axel Haarmann, Patrick Oschmann, Veit Rothhammer, Christian Geis, Markus C. Kowarik, Peter Kern, Matthias Grothe, Heike Stephanik, Klemens Angstwurm, Frank Hoffmann, Ulrike Wallwitz, Brigitte Wildemann, Sven Jarius, Jan-Patrick Stellmann, Thivya Pakeerathan, Carolin Schwake, Ilya Ayzenberg, Ingo Kleiter, Katinka Fischer, Orhan Aktas, Marius Ringelstein, Vivien Häußler, Corinna Trebst, Martin W. Hümmert; on behalf of the Neuromyelitis optica Study Group (NEMOS)

Supplementary Table 1. Overview of previous studies about patients with late-onset NMOSD

Author	Year	Country	N	Late-onset / early-onset NMOSD)	Serostatus	Clinical outcome of LO-NMOSD	Attack and/ or long-term therapy
**Collongues et.al**	2014	France, Germany, Turkey and United Kingdom	430	108 LO-NMO/NMOSD	82 AQP4-IgG positive LO-NMOSD	LO-NMO/ LO-NMOSD patients suffered motor impairment and death	89% received IST
**Contentti et.al**	2020	LATAM (Argentina, Brazil and Venezuela)	140	24 LO-NMOSD / 116 EO-NMOSD	16 AQP4-IgG positive LO-NMOSD / 73 AQP4-IgG positive EO-NMOSD	Severe disability at early disease stage, and higher EDSS at last follow up, shorter time to reach EDSS 4	No differences long-term IST and acute treatment
**Hu et al.**	2020	China	298	134 LO-NMOSD / 164 EO-NMOSD	113 AQP4-IgG positive LO-NMOSD / 134 AQP4-IgG positive EO-NMOSD	Higher EDSS score at last follow- up	EO-NMOSD used more long-term IST, no differences in acute attack therapy
**Mao et al.**	2015	China	60	30 LO-NMOSD /30 EO-NMOSD	AQP4-IgG positive patients only	Higher nadir EDSS, shorter time to EDSS 4 and higher EDSS at last follow up	No differences in IST
**Min et.al**	2022	China	50	22 LO-NMOSD / 28 EO-NMOSD	AQP4-IgG positive patients only	LO-NMOSD patients had a worse prognosis	Not analyzed
**Papathanasiou et.al**	2021	United Kingdom	52	26 LO-NMOSD / 26 EO-NMOSD	AQP4-IgG positive patients only	Higher EDSS at last follow up	No differences in acute and long-term IST
**Santos et.al**	2022	Portuguese	180	35 LO - NMOSD / 145 EO-NMOSD	32 AQP4-IgG positive LO-NMOSD and 81AQP4-IgG positive EO-NMOSD	Higher EDSS at last follow up	No differences long-term IST and acute treatment

**Seok et.al**	2017	Korea	147	45 LO-NMOSD / 102 EO-NMOSD	AQP4-IgG positive patients only	Positive correlation between age at onset and EDSS at last follow up	No differences in IST use, EO-NMOSD used more oral prednisolone and previous beta-interferon treatment ,
**Sepulveda et.al**	2019	Spain	238	69 LO-NMOSD / 169 EO-NMOSD	60 AQP4-IgG positive LO-NMOSD / 133 AQP4-IgG positive EO-NMOSD	Higher EDSS at last follow up	EO used more long-term IST
**Wang et.al**	2022	China	490	122 LO-NMOSD / 368-NMOSD EO	101 AQP4-IgG LO-NMOSD	Higher EDSS score, and worse prognosis, age at onset predicts blindness and motor dysfunction	No differences long-term IST and acute treatment
**Zhang et.al**	2017	China	142	83 EO-NMOSD and 59 LO-NMOSD	52 AQP4-IgG positive LO-NMOSD LO and 70 AQP4-IgG positive EO-NMOSD	LO-NMOSD patients suffered more motor disability, EO-NMOSD patients more visual disability	Not analyzed

Abbreviations: NMOSD= Neuromyelitis optica spectrum disorders, LO= late-onset, EO= early-onset, EDSS= Expanded Disability Status Scale.; IST= immunosuppressant treatment

[1]The table lists key points of the largest and most relevant studies for the reported investigation.

[2]Aspects relate exclusively for LO-NMOSD patients

Supplementary Table 2.1. Demographic characteristics, attack type at disease onset and comorbidities categorized by age at disease onset for AQP4-IgG positive NMOSD

	Available n	NMOSD (N=381)	LO-NMOSD (n=144)	EO-NMOSD (n=237)	*p*-value
**Demography**					
Female, n (%)	380	336 (88.4%)	117 (81.3%)	219 (92.8%)	**<0.001**
Age at onset, median (range), y	381	43 (6-84)	59 (50-84)	35 (6-49)	**<0.001**
Age at diagnosis, median (range), y	381	49 (6-85)	61 (50-84)	41 (6-68)	**<0.001**
Age at database entry, median (range), y	277	53 (18-85)	65 (51-85)	45 (18-77)	**<0.001**
Time to diagnosis, median (range)[2], y	378	1 (0-41)	0 (0-18)	1 (0-41)	**<0.001**
Follow-up time, median (range), y	294	1 (0-14)	1 (0-7)	2 (0-14)	**0.021**
Disease duration, median (range), y[3]	295	8 (0-52)	5.5 (0-27)	9 (0-52)	**<0.001**
**Ethnicity, n (%)**	363				0.279
Whites		333 (91.7%)	128 (94.8)	205 (89.9%)	
Asian		7 (1.9%)	2 (1.5%)	5 (2.2%)	
Arabic		8 (2.2%)	1 (0.7%)	7 (3%)	
Latin		3 (0.8%)	2 (1.5%)	1 (0.4%)	
African		9 (0%)	2 (1.5%)	7 (3.1%)	
Other		3 (1.3%)	0 (0%)	3 (0.8%)	
**Attack type at disease onset, n (%)**					
Optic neuritis	365	135 (36.9%)	39 (28.5%)	96 (42.1%)	**0.007**
Myelitis	365	169 (46.3%)	79 (57.7%)	90 (39.5%)	**0.001**
Optic neuritis and myelitis	365	15 (4.1%)	5 (3.6%)	10 (4.4%)	0.866
Brainstem encephalitis	365	7 (1.9%)	1 (0.7%)	6 (2.6%)	0.097
Area postrema syndrome	365	9 (2.5%)	2 (1.5%)	7 (3.1%)	0.459
Diencephalic syndrome	365	0	0	0	NA
Cerebral syndrome	365	2 (0.5%)	1 (0.7%)	1 (0.4%)	0.715
Multiple symptoms	365	15 (4.1%)	5 (3.6%)	10 (4.4%)	0.946
Other	365	13 (3.6%)	5 (3.6%)	8 (3.5%)	0.944
**Comorbidities, n (%)**					
Autoimmune comorbidities[4]	347	129 (37.2%)	42 (32.8%)	87 (39.1%)	0.209
Hashimoto thyroiditis		37 (28.7%)	12 (28.6%)	25 (28.7%)	
SLE		33 (25.6%)	7 (16.7%)	26 29.9	
Sjögren`s syndrome		20 (15.5%)	5 (11.9%)	15 (17.2%)	
Myasthenia gravis		12 (9.3%)	4 (9.5%)	8 (9.2%)	
Rheumatoid arthritis		8 (6.2%)	7 (16.7%)	1 (1.1%)	

Other [5]		63 (48.8%)	31 (73.8%)	32 (36.8%)	
Non autoimmune comorbidities	343	200 (58.3%)	97 (73.5%)	103 (48.8%)	**<0.001**
Cardiovascular diseases	343	78 (22.7%)	54 (40.9%)	24 (11.4%)	**<0.001**
Oncological diseases	343	34 (9.9%)	24 (18.2%)	10 (4.8%)	**<0.001**

Abbreviations.: AQP4-IgG= aquaporin-4 immunoglobulin G; MOG-IgG= myelin oligodendrocyte glycoprotein immunoglobulin G; NMOSD= Neuromyelitis optica spectrum disorders, LO= late-onset, EO= early-onset; y= years; n/a= not available, SLE= Systemic Lupus Erythematosus . NA= not available

Supplementary Table 2.2. Demographic characteristics, attack type at disease onset and comorbidities categorized by age at disease onset for AQP4-IgG negative NMOSD patients

	Available n	NMOSD (N=65)	LO-NMOSD (n=9)	EO-NMOSD (n=56)	*p*-value
**Demography**					
Female, n (%)	65	35 (53.8%)	6 (66.7%)	29 (51.8%)	0.406
Age at onset, median (range), y	65	34 (5-70)	56 (51-70)	32 (5-49)	**0.002**
Age at diagnosis, median (range), y	65	39 (12-71)	56 (51-70)	36 (12-50)	**<0.001**
Age at database entry, median (range), y	40	45.5 (23-72)	60 (56-72)	42 (23-56)	**0.006**
Time to diagnosis, median (range)[2], y	65	2 (0-22)	1 (0-2)	2.5 (0-22)	**0.014**
Follow-up time, median (range), y	51	1 (0-13)	0.5 (0-2)	1 (0-13)	0.701
Disease duration, median (range), y[3]	51	8 (1-28)	4 (0-10)	9 (0-28)	0.701
**Ethnicity, n (%)**	64				0.773
Whites		61 (95.3%)	9 (100%)	52 (94.5%)	
Asian		0 (0%)	0(0%)	0 (0%)	
Arabic		2 (3.1%)	0 (0%)	2 (3.6%)	
Latin		1 (1.6%)	0(0%)	1 (1.8%)	
African		0 (0%)	0 (0%)	0 (0%)	
Other		0 (0%)	0 (0%)	0 (0%)	
**Attack type at disease onset, n (%)**					
Optic neuritis	63	25 (39.7%)	1 (11.1%)	24 (44.4)	0.072
Myelitis	63	23 (36.5%)	6 (66.7%)	17 (31.5%)	**0.042**
Optic neuritis and myelitis	63	3 (4.8%)	0 (0%)	3 (5.6%)	0.496
Brainstem encephalitis	63	3 (4.8%)	0 (0%)	3 (5.6%)	0.496
Area postrema syndrome	63	0	0	0	NA
Diencephalic syndrome	63	0	0	0	NA
Cerebral syndrome	63	0	0	0	NA
Multiple symptoms	63	3 (4.8%)	1 (11.1%)	2 (3.7%)	0.527
Other	63	6 (9.5%)	1 (11.1%)	5 (9.3%)	0.861
**Comorbidities, n (%)**					

Autoimmune comorbidities[4]	65	5 (8.2%)	0 (0%)	5 (9.6%)	0.332
Hashimoto thyroiditis		3(60%)	0 (0%)	3 (60%)	
SLE		1 (20%)	0 (0%)	1 (20%)	
Other [5]		1 (20%)	0 (0%)	1 (20%)	
Non autoimmune comorbidities	61	33 (54.1%)	4 (50%)	29 (54.7%)	0.803
Cardiovascular diseases	61	9 (14.8%)	2 (25.0%)	7 (13.2%)	0.391
Oncological diseases	61	3 (4.9%)	1 (12.5%)	2 (3.8%)	0.287

Abbreviations.: AQP4-IgG= aquaporin-4 immunoglobulin G; MOG-IgG= myelin oligodendrocyte glycoprotein immunoglobulin G; NMOSD= Neuromyelitis optica spectrum disorders, LO= late-onset, EO= early-onset; y= years; n/a= not available, SLE= Systemic Lupus Erythematosus . NA= not available

[1]Percentages may not add exactly to 100% because of rounding. [2]Time between onset and NMOSD diagnosis in years. [3]Time between onset and last follow up. [4]Each autoimmune comorbidity was considered individually.[5]Other: Type 1 diabetes mellitus, psoriasis, autoimmune hepatitis, vitiligo, ankylosing spondylitis, Crohn´s disease, Grave´s disease, celiac disease, idiopathic thrombocytopenic purpura, uveitis, iritis, primary biliary cirrhosis, scleroderma.

Supplementary Table 3.1. Detailed attack data of AQP4-IgG positive NMOSD patients, categorized by age at disease onset

	**Available n**	**NMOSD**	**LO-NMOSD**	**EO-NMOSD**	***p*-value**
**AAR, mean (SD)**[1]	184				
Total attacks		0.53 (0.43)	0.51 (0.52)	0.54 (0.39)	0.292
Myelitis attacks		0.37 (0.31)	0.38 (0.28)	0.37 (0.33)	0.425
Optic neuritis attacks		0.27 (0.36)	0.37 (0.65)	0.24 (0.25)	0.553
Optic neuritis and myelitis attacks		0.10 (0.06)	0.10 (0.01)	0.20 (0.07)	0.583
Monophasic course, n (%)	291	24 (8.2%)	13 (13.1%)	11 (5.7%)	**0.030**
Time to second attack, months (median, range)	266	11.5 (1-491)	8 (1-220)	13 (1-491)	0.086
**RAW**[2]**, median (IQR)**					
RAW at all clinical attacks	161	1.5 (-4-9)	3 (0-9)	0.5 (-4-8)	**<0.001**
RAW[3] at onset	86	3 (0-9)	4 (0-9)	2 (0-8)	**<0.001**
**IVMP therapy, n (%)**	1102[3]				
IVMP alone		874 (79.3%)	199 (72.4%)	675 (81.6%)	**0.011**
IVMP with PE/IA		228 (20.7%)	76 (27.6%)	152 (18.4%)	**0.001**
Total dose of IVMP mg/attack, mean (SD)	687	5184 (3159)	6170 (3630)	4919 (2962)	0.265
**Apheresis therapy, n (%)**	319				
Plasma exchange		242 (75.9%)	64 (73.6%)	178 (76.4%)	0.621
Immunoadsorption		50 (15.6%)	15 (17.2%)	35 (15.0%)	0.621
Plasma exchange + immunoadsorption		27 (8.5%)	8 (9.2%)	19 (8.2%)	
Apheresis therapy cycles, mean (SD)	273	6.82 (2.5)	6.80 (2.52)	6.83 (2.53)	0.901

Abbreviations.: NMOSD= Neuromyelitis optica spectrum disorder; LO= late-onset; EO= early-onset; EDSS= Expanded Disability Status Scale; ARR= Annualized Attack Rate; PE= Plasmaexchange; IA=Immunoabsorption; IVMP= Intravenous methylprednisolone pulse; IQR= interquartile range; SD= standard deviation; mg= milligram; RAW= relapse associated worsening

[1]Annualized attack rate (Number of total attacks divided by disease duration), symptom specific stratification. Only patients with at least 12 month of follow-up time were included. [2]RAW: EDSS difference between basal EDSS before attack and EDSS ≥ 90 days after attack, if no further attack occurred. [3]RAW after disease onset.[4] Intravenous methylprednisolone and apheresis therapy (plasma exchange and/or immunoadsorption), subgroup specific for late- and early-onset.

Supplementary Table 3.2. Detailed attack data of AQP4-IgG negative NMOSD patients, categorized by age at disease onset

	Available n	NMOSD	LO-NMOSD	EO-NMOSD	*p*-value
**AAR, mean (SD)[1]**	27				
Total attacks		0.53 (0.43)	0.46 (0.19)	0.54 (0.45)	0.799
Myelitis attacks		0.25 (0.18)	0.22 (0.16)	0.25 (0.19)	0.952
Optic neuritis attacks		0.27 (0.33)	0.15 (0.07)	0.28 (0.34)	0.947
Optic neuritis and myelitis attacks		0.13 (0.08)	0.3 (NA)	0.11 (0.05)	0.200
Monophasic course, n (%)	49	3 (6.1%)	0 (0%)	3 (7.1%)	0.466
Time to second attack, months (median, range)	44	11.5 (1-262)	12 (1-88)	11.5 (1-262)	0.660
**RAW[2], median (IQR)**					
RAW at all clinical attacks	23	1.5 (-0.5-6)	2 (2-2)	1.25 (-0.5-6)	0.391
RAW[3] at onset	8	3.75 (1.5-6)	NA (NA)	3.75 (1.5-6)	NA
**IVMP therapy, n (%)**	152[3]				
IVMP alone		140 (92.1%)	9 (69.2%)	131 (94.2%)	**0.011**
IVMP with PE/IA		12 (7.9%)	4 (30.8%)	8 (5.8%)	**0.011**
Total dose of IVMP mg/attack, mean (SD)	88	5214 (2886)	5470 (2931)	5177 (2931)	0.265
**Apheresis therapy, n (%)**	19				
Plasma exchange		16 (84.2%)	6 (66.7%)	10 (100%)	0.087
Immunoadsorption		3 (15.6%)	3 (33.3%)	0 (0%)	0.087
Apheresis therapy cycles, mean (SD)	14	5.3 (1.4)	5 (0.63)	5.50 (1.85)	0.391

Abbreviations.: NMOSD= Neuromyelitis optica spectrum disorder; LO= late-onset; EO= early-onset; EDSS= Expanded Disability Status Scale; ARR= Annualized Attack Rate; PE= Plasmaexchange; IA=Immunoabsorption; IVMP= Intravenous methylprednisolone pulse; IQR= interquartile range; SD= standard deviation; mg= milligram; RAW= relapse associated worsening

[1]Annualized attack rate (Number of total attacks divided by disease duration), symptom specific stratification. Only patients with at least 12 month of follow-up time were included. [2]RAW: EDSS difference between basal EDSS before attack and EDSS ≥ 90 days after attack, if no further attack occurred. [3]RAW after disease onset.[4] Intravenous methylprednisolone and apheresis therapy (plasma exchange and/or immunoadsorption), subgroup specific for late- and early-onset.

Supplementary Table 4.1. Odds ratios for full recovery based on different attack types and treatment modalities between AQP4-IgG positive LO- and EO-NMOSD patients in generalized estimating equations (GEE) analysis.

	**OR (95% CI)**[2]	***p*-value**[1]
**Recovery**		
**All attacks**	0.465 (0.298 - 0.728)	**<0.001**

Supplementary Table 4.2. Odds ratios for full recovery based on different attack types and treatment modalities between AQP4-IgG negative LO- and EO-NMOSD patients in generalized estimating equations (GEE) analysis.

	**OR (95% CI)**[2]	***p*-value**[1]
**Recovery**		
**All attacks**	0.241 (0.065 - 0.899)	**0.034**

Supplementary Table 5.1. Detailed data on long-term immunotherapies, categorized by age at disease onset for AQP4-IgG positive NMOSD patients

	Available n	NMOSD	LO-NMOSD	EO-NMOSD	p-value
**Immunotherapy**					
Immunotherapy (yes vs. no/unknown)	381	344 (90.3%)	126 (87.5%)	218 (92.0%)	0.214
Switch in immunotherapy (yes vs. no/unknown)	325	159 (48.9%)	49 (41.2%)	110 (53.5)	**0.038**
Time from onset to immunotherapy, months (median, range)[1]	313	11 (0-562)	6 (0-210)	16 (0-562)	0.012
**First line immunotherapy[1]**	337				
B-cell depletion		146 (43.3%)	56 (45.5%)	90 (42.1%)	0.536
Classical immunosuppressants		113 (33.5%)	42 (34.1%)	71 (33.2%)	0.856
IL-6-receptor inhibition		2 (0.6%)	0 (%)	2 (0.9%)	0.282
Complement inhibition		14 (4.1%)	9 (7.3%)	5 (2.3%)	**0.044**
Other		53 (15.4%)	11 (8.9%)	42 (19.6%)	0.012
**Immunotherapy used at the last follow-up**	335				
B-cell depletion		205 (61.2%)	77 (62.1%)	128 (60.7%)	0.795
Classical immunosuppressants		67 (20.0%)	25 (20.2%)	42 (19.9%)	0.955
IL-6-receptor inhibition		33 (9.9%)	9 (7.3%)	24 (11.4%)	0.222
Complement inhibition		24 (7.2%)	13 (10.5%)	11 (5.2%)	0.081
Other		6 (1.8%)	0 (0%)	6 (2.8%)	0.058
Post-treatment ARR[2] (mean, SD)	180	0.31 (0.53)	0.23 (0.45)	0.35 (0.26)	**0.013**
**Attack-free under RTX first-line[3]**					
Attack-free after 6 mo. n, (%)	113	88 (77.9%)	33 (78.6%)	55 (77.5%)	0.891
Attack free after 12 mo. n, (%)	112	83 (74.1%)	33 (78.6%)	50 (71.4%)	0.403
Attack free after 36 mo. n, (%)	74	42 (56.8%)	16 (57.1%)	26 (56.5%)	0.958
Attack free after 60 mo. n, (%)	54	22 (40.7%)	9 (45.0%)	13 (38.1%)	0.625
**Attack-free under RTX any line[4]**					
Attack free after 6 mo. n, (%)	169	138 (81.7%)	52 (85.2%)	86 (79.6%)	0.365
Attack free after 12 mo. n, (%)	162	125 (77.2%)	51 (85.0%)	74 (72.5%)	0.068
Attack free after 36 mo. n, (%)	115	71 (61.7%)	28 (65.1%)	43 (59.7%)	0.565

Attack free after 60 mo. n, (%)	88	40 (45.5%)	14 (46.7%)	26 (44.8%)	0.870

Abbreviations.: NMOSD= Neuromyelitis optica spectrum disorder; LO= late-onset; EO= early-onset; RTX= rituximab, AZA= azathioprine, MTX=metothrexate, MMF= mycophenolate mofetil, IL-6= interleukin-6, ARR= annualized attack rate

[1]Immunotherapy: B-cell depletion (RTX/inebilizumab), classical immunosuppressants (AZA, MMF, MTX, oral steroids), IL6-receptor inhibition (tocilizumab/satralizumab), Complement inhibition (eculizumab), Other (glatiramer acetate, interferon beta, mitoxantrone, fingolimod, alemtuzumab, natalizumab, dimethyl fumarate, intravenous immunoglobulins, cyclophosphamide).

[2]Post treatment ARR = Number of attacks after initiation of NMOSD therapy divided by the time between initiation and last follow-up.

[3]Frequency of clinical stable course after 6, 12, 36, and 60 month after first line therapy with RTX.

[4]Frequency of clinical stable course after 6, 12, 36, and 60 month after treatment during the course of the disease with RTX.

Supplementary Table 5.2. Detailed data on long-term immunotherapies, categorized by age at disease

onset for AQP4-IgG negative NMOSD patients

	Available n	NMOSD	LO-NMOSD	EO-NMOSD	p-value
**Immunotherapy**					
Immunotherapy (yes vs. no/unknown)	65	59 (90.8%)	8 (88.9%)	51 (91.1%)	0.843
Switch in immunotherapy (yes vs. no/unknown)	55	17 (30.9%)	4 (50.0%)	13 (27.7%)	0.206
Time from onset to immunotherapy, months (median, range)[2]	45	15 (0-190)	12 (1-83)	16.5 (0-190)	0.949
**First line immunotherapy[1]**	57				
B-cell depletion		26 (45.6%)	7 (87.5%)	19 (38.8%)	**0.018**
Classical immunosuppressants		19 (33.3%)	1 (12.5%)	18 (36.7%)	0.178
IL-6-receptor inhibition		0 (0%)	0 (0%)	0 (0%)	NA
Complement inhibition		0 (0%)	0 (0%)	0 (0%)	NA
Other		12 (21.1%)	0 (0%)	12 (24.5%)	0.178
**Immunotherapy used at the last follow-up**	55				
B-cell depletion		36 (65.5%)	7 (87.5%)	29 (61.7%)	0.156
Classical immunosuppressants		16 (29.1%)	1 (12.5%)	15 (31.9%)	0.264
IL-6-receptor inhibition		1 (1.8%)	0 (0%)	1 (2.1%)	0.677

Complement inhibition		0 (0%)	0 (0%)	0 (0%)	NA
Other		2 (3.6%)	0 (0%)	2 (4.3%)	0.552
Post-treatment ARR[2] (mean, SD)	27	0.35 (0.23)	0.43 (0.25)	0.35 (0.22)	0.914
**Attack-free under RTX first-line[3]**					
Attack-free after 6 mo. n, (%)	15	12 (80.0%)	2 (66.7%)	10 (83.3%)	0.519
Attack free after 12 mo. n, (%)	14	9 (64.3%)	1 (33.3%)	8 (72.7%)	0.207
Attack free after 36 mo. n, (%)	12	7 (58.3%)	1 (50.0%)	6 (60.0%)	0.958
Attack free after 60 mo. n, (%)	7	3 (42.9%)	0 (0%)	3 (50.0%)	0.250
**Attack-free under RTX any line[4]**					
Attack free after 6 mo. n, (%)	25	20 (80.0%)	2 (66.7%)	18 (81.8%)	0.538
Attack free after 12 mo. n, (%)	23	18 (78.3%)	2 (66.7%)	16 (80.0%)	0.602
Attack free after 36 mo. n, (%)	20	12 (60.0%)	1 (50.0%)	11 (61.1%)	0.761
Attack free after 60 mo. n, (%)	11	6 (54.5%)	0 (0%)	6 (60.0%)	0.251

Abbreviations.: NMOSD= Neuromyelitis optica spectrum disorder; LO= late-onset; EO= early-onset; RTX= rituximab, AZA= azathioprine, MTX=metothrexate, MMF= mycophenolate mofetil, IL-6= interleukin-6, ARR= annualized attack rate

[1]Immunotherapy: B-cell depletion (RTX/inebilizumab), classical immunosuppressants (AZA, MMF, MTX, oral steroids), IL6-receptor inhibition (tocilizumab/satralizumab), Complement inhibition (eculizumab), Other (glatiramer acetate, interferon beta, mitoxantrone, fingolimod, alemtuzumab, natalizumab, dimethyl fumarate, intravenous immunoglobulins, cyclophosphamide).

[2]Post treatment ARR = Number of attacks after initiation of NMOSD therapy divided by the time between initiation and last follow-up.

[3]Frequency of clinical stable course after 6, 12, 36, and 60 month after first line therapy with RTX.

[4]Frequency of clinical stable course after 6, 12, 36, and 60 month after treatment during the course of the disease with RTX.

Supplementary Table 6. Generalized linear mixed model (GLMM) analysis of full recovery[1]

	p-value	**OR**	**95% CI**
**Age at attack**			
All attacks [2]	**<0.001**	0.968	0.956 – 0.979
Isolated myelitis[3]	**0.005**	0.975	0.957 – 0.992
Isolated optic neuritis[4]	**0.015**	0.972	0.950 – 0.995
Abbreviations: GLMM= Generalized linear mixed model; OR= odds ratio; CI= confidence interval			

[1] OR were adjusted for age at attack, sex (female vs. male), treatment type (IVMP vs. IVMP + apheresis) and diagnosis (AQP4-IgG positive NMOSD vs AQP4-IgG and MOG-IgG negative NMOSD)- Values are shown for age at attac. Other independent values are listed below.

[2] Sex (female vs. male): OR = 2.368, 95% CI: 1.272–4.407, p = 0.007; Treatment type (IVMP vs. IVMP + PLEX): OR = 4.891, 95% CI: 2.686–8.908, p < 0.001; Diagnosis (AQP4-IgG positive vs. negative): OR = 0.618, 95% CI: 0.378–1.009, p = 0.054

[3] Sex (female vs. male): OR = 2.930, 95% CI: 1.035–8.294, p = 0.043; Treatment type (IVMP vs. IVMP + PLEX): OR = 4.149, 95% CI: 1.816–9.481, p < 0.001; Diagnosis (AQP4-IgG positive vs. negative): OR = 0.511, 95% CI: 0.215–1.214, p = 0.128

[4] Sex (female vs. male): OR = 2.097, 95% CI: 0.663–6.631, p = 0.207; Treatment type (IVMP vs. IVMP + PLEX): OR = 5.146, 95% CI: 1.989–13.316, p = 0.001; Diagnosis (AQP4-IgG positive vs. negative): OR = 0.681, 95% CI: 0.259–1.794, p = 0.436

eTable 7. Coinvestigators

**Appendix 1** Coinvestigators			
**Name**	**Location**	**Role**	**Contribution**
Simone Tauber, MD	University Hospital Aachen	Site Investigator	Organizational support
Verena Steuerwald, MD	University Hospital Augsburg	Site Investigator	Organizational support
Julia Gutbrod, MD	University Hospital Augsburg	Site Investigator	Organizational support
Mathias Buttmann, MD	Caritas-Krankenhaus, Bad Mergentheim	Site Investigator	Organizational support
Peter Luedemann, MD	Agaplesion EV. Bathildiskrankenhaus, Bad Pyrmont	Site Investigator	Organizational support
Ingo Kleiter, MD	Marianne-Strauss-Klinik, Berg	Site Investigator	Organizational support
Ankelien Duchow, MD	Charité University, Berlin	Site Investigator	Organizational support
Susanna Asseyer, MD	Charité University, Berlin	Site Investigator	Organizational support
Maria Hastermann, MD	Charité University, Berlin	Site Investigator	Organizational support
Florence Pache, MD	Charité University, Berlin	Site Investigator	Organizational support
Carsten Finke, MD	Charité University, Berlin	Site Investigator	Organizational support
Anna Gahlen, MD	St. Josef-Hospital, Ruhr University Bochum	Site Investigator	Organizational support
Theodoros Ladopoulus, MD	St. Josef-Hospital, Ruhr University Bochum	Site Investigator	Organizational support
Mathias von Mering, MD	Klinikum Bremen Nord	Site Investigator	Organizational support
Kevin Rostasy, MD	Vestische Kinder und Jugendklinik Datteln	Site Investigator	Organizational support

Ulrich Hofstadt-van Oy, MD	Klinikum Westfalen, Dortmund	Site Investigator	Organizational support
Andreas Steinbrecher, MD	Helios Klinikum, Erfurt	Site Investigator	Organizational support
Christoph Kleinschnitz, MD	University Hospital, Essen, Germany	Site Investigator	Organizational support
Thanos Tsaktanis, MD	University Hospital Erlangen	Site Investigator	Organizational support
Sebastian Rauer, MD	University Hospital Freiburg	Site Investigator	Organizational support
Christoph Mayer, MD	Neurologische Gemeinschaftspraxis am Kaiserplatz	Site Investigator	Organizational support
Steffen Pfeuffer, MD	University Hospital Giessen	Site Investigator	Organizational support
Kersten Guthke, MD	Städtisches Klinikum, Goerlitz	Site Investigator	Organizational support
Kersten Guthke, MD	Städtisches Klinikum, Goerlitz	Site Investigator	Organizational support
Imke Metz, MD	University Hospital Göttingen	Site Investigator	Organizational support
Leila Husseini, MD	University Hospital Göttingen	Site Investigator	Organizational support
Achim Gass, MD	University Hospital Mannheim	Site Investigator	Organizational support
Annette Walter, MD	Klinikum Herford	Site Investigator	Organizational support
Mathias Fousse, MD	University Hospital Saarland	Site Investigator	Organizational support
Matthias Schwab, MD	University Hospital Jena	Site Investigator	Organizational support
Frank Leypoldt, MD	University Hospital Schleswig Holstein, Campus Kiel	Site Investigator	Organizational support
Klarissa Stuerner, MD	University Hospital Schleswig Holstein, Campus Kiel	Site Investigator	Organizational support
Mariella Herfurth, MD	University Hospital Leipzig	Site Investigator	Organizational support
Wolfgang Köhler, MD	University Hospital Leipzig	Site Investigator	Organizational support
Muriel Stoppe, MD	University Hospital Leipzig	Site Investigator	Organizational support
Klaus-Peter Wandinger, MD	University Hospital Schleswig Holstein, Campus Lübeck	Site Investigator	Organizational support
Sergiu Groppa, MD	University Hospital Mainz	Site Investigator	Organizational support
Stefan Bittner, MD	University Hospital Mainz	Site Investigator	Organizational support
Frauke Zipp, MD	University Hospital Mainz	Site Investigator	Organizational support

Moritz Förster, MD	Maria-Hilf Hospital, Mönchengladbach	Site Investigator	Organizational support
Philipp Albrecht, MD	Maria-Hilf Hospital, Mönchengladbach	Site Investigator	Organizational support
Bernhard Hemmer, MD	Technical University of Munich	Site Investigator	Organizational support
Martin Marziniak, MD	kbo - Isar - Amper-Klinikum München Ost	Site Investigator	Organizational support
Heinz Wiendl, MD	University Hospital Münster	Site Investigator	Organizational support
Markus Krumbholz, MD	Medizinische Hochschule Brandenburg, Rüdersdorf	Site Investigator	Organizational support
Pawel Kermer, MD	Nordwest Krankenhaus, Sanderbusch	Site Investigator	Organizational support
Oliver Neuhaus, MD	SRH Klinik Landkreis, Sigmaringen	Site Investigator	Organizational support
Alexander Stefanou, MD	Klinikum der Landeshauptstadt, Katharinenhospital, Stuttgart	Site Investigator	Organizational support
Arthur Melms, MD	Facharztpraxis für Neurologie und Psychiatrie	Site Investigator	Organizational support
Jasmin Naumann, MD	Knappschaftsklinikum Saar, Sulzbach	Site Investigator	Organizational support
Stefanie Behnke, MD	Knappschaftsklinikum Saar, Sulzbach	Site Investigator	Organizational support
Karsten Kern, MD	Knappschaftsklinikum Saar, Sulzbach	Site Investigator	Organizational support
Arne Riedlinger, MD	Asklepios Fachklinikum, Teupitz	Site Investigator	Organizational support
Patricia Schwarz, MD	University Hospital Tübingen	Site Investigator	Organizational support
Christoph Ruschil, MD	University Hospital Tübingen	Site Investigator	Organizational support
Ulf Ziemann, MD	University Hospital Tübingen	Site Investigator	Organizational support
Martin Liebetrau, MD	St. Josef Hospital, Wiesbaden	Site Investigator	Organizational support
Axel Haarman, MD	University Hospital Würzburg	Site Investigator	Organizational support
Claudia Sommer, MD	University Hospital Würzburg	Site Investigator	Organizational support
Marina Herwerth, MD	University Hospital Zurich	Site Investigator	Organizational support
Anne-Katrin Proebstel, MD	University Hospital Basel	Site Investigator	Organizational support
Tradite Neziraj, MD	University Hospital Basel	Site Investigator	Organizational support

Roxanne Pretzsch, MD	University Hospital Basel	Site Investigator	Organizational support
